# Performance of Down syndrome subjects during a coincident timing task

**DOI:** 10.1186/1755-7682-6-15

**Published:** 2013-04-24

**Authors:** Camila Torriani-Pasin, Giordano MG Bonuzzi, Marcos AA Soares, Gisele L Antunes, Gisele CS Palma, Carlos BM Monteiro, Luiz Carlos de Abreu, Vitor E Valenti, Alaércio Perotti Junior, Rubens Wajnsztejn, Umberto C Corrêa

**Affiliations:** 1 Motor Behavior Laboratory (Lacom), School of Physical Education and Sport, University of Sao Paulo, Av. Prof. Mello de Morais, 65, Sao Paulo, SP, 05508-030, Brazil; 2 School of Arts, Sciences and Humanities, University of Sao Paulo, Av. Arlindo Béttio, 1000, Sao Paulo, SP, 03828-000, Brazil; 3 Laboratory of Scientific Writing, Department of Morphology and Physiology, School of Medicine of ABC, Av. Príncipe de Gales, 821, Santo Andre, SP, 09060-650, Brazil; 4 Department of Speech Language and Hearing Therapy, Faculty of Philosophy and Sciences, UNESP, Av. Hygino Muzzi Filho, Marilia, SP, 737.17.525-900, Brazil

**Keywords:** Growth & development, Down syndrome, Motor activity, Task performance and analysis

## Abstract

**Background:**

The time synchronization is a very important ability for the acquisition and performance of motor skills that generate the need to adapt the actions of body segments to external events of the environment that are changing their position in space. Down Syndrome (DS) individuals may present some deficits to perform tasks with synchronization demand. We aimed to investigate the performance of individuals with DS in a simple Coincident Timing task.

**Method:**

32 individuals were divided into 2 groups: the Down syndrome group (DSG) comprised of 16 individuals with average age of 20 (+/− 5 years old), and a control group (CG) comprised of 16 individuals of the same age. All individuals performed the Simple Timing (ST) task and their performance was measured in milliseconds. The study was conducted in a single phase with the execution of 20 consecutive trials for each participant.

**Results:**

There was a significant difference in the intergroup analysis for the accuracy adjustment - Absolute Error (Z = 3.656, p = 0.001); and for the performance consistence - Variable Error (Z = 2.939, p = 0.003).

**Conclusion:**

DS individuals have more difficulty in integrating the motor action to an external stimulus and they also present more inconsistence in performance. Both groups presented the same tendency to delay their motor responses.

## Background

Over the years, many studies have shown increasing interest in investigating how the individuals with physical and mental disabilities perform and acquire movements, including those with Down Syndrome (DS) [1]. The main research questions that have been frequently asked in these studies are on: (a) whether such movements can be considered normal or abnormal [2,3], (b) understanding and explaining the mechanisms and processes underlying the acquisition of these movements [4,5], and (c) raising subsidies to elaborate new programs for physical practice and therapies [1].

The DS has serious implications in the neural, physiological, and biomechanical systems, which causes the individuals to present some peculiarities, such as is “clumsiness,” that is characterized by movements profile as slow movements with impaired coordination and increased rates of failure, reduced reaction times, decreased muscle tone, impaired control of timing and difficulty to modulate actions under changing task conditions, such as grasp forces and postural responses [6-12]. Alternative explanations to justify these findings have suggested a different brain activation of the motor response in DS individuals [13].

The atypical patterns of brain organization could be responsible for many cognitive features that generate certain difficulty in organization, perception, and motor response [1]. The movement features of DS individuals can be partially attributed to the structural characteristics, such as the operation of the brain stem and size of cerebellum [13,14]. The biomechanical profile of DS is an issue that may be attributed as a result of these atypical patterns of brain organization, there is evidence that the individuals with DS present a deficit in performing tasks with predominance in perception requirements, mainly in tasks that demand time synchronization [15-18].

According to Schmidt and Wrisberg (2001) [19] the time synchronization is a very important ability for the acquisition and performance of motor skills that generate the need to adapt the actions of body segments to external events of the environment that are changing their position in space. These actions have been given several names over the years, such as: coincident anticipation [19], temporal organization of anticipation [19], timing and anticipation [20,21], coincident timing [22], and anticipatory timing [23]. In this study, we used the term coincident timing as it is one of the most used in the literature.

In the area of motor learning, the coincident timing has been investigated in healthy subjects during several aspects, for example, the variability of practice [24], stimulus speed [25], age [26], gender [27], level of tasks complexity [28], level of skill [29], knowledge of results [30]. However, when analyzing the literature mentioned above, there are no studies designed for DS individuals involving coincident timing tasks.

It is important to carry out studies that contribute to the comprehension of the motor skill of people with DS [18], especially with respect to the performance of tasks that require coincident timing. Therefore, the aim of this study was to investigate the performance of individuals with DS in a simple Coincident Timing task.

## Method

### Participants

32 individuals participated in this study and were divided into 2 groups: the Down syndrome group (DSG) comprised of 16 individuals with average age of 20 (+/− 5 years old), classified into slight and moderate, according to the International Classification of Functioning, Disability, and Health (ICF); and a control group (CG) comprised of 16 individuals of the same age. We excluded subjects who were not able to perform the tests. The participation in the experiment was conditioned to the signing of the informed consent form by those responsible. This study was approved by the Independent Ethics Committee of the University with protocol number 942/2010.

Table 1 presents data regarding the severity of impairment.

**Table 1:** Sample characterization regarding the experimental group

	d1550*	d1551*	d160*	d177*	d2101*	d310*	**TOTAL**
**EG/ICF mild**	4	5	5	5	5	5	8
**EG/ICF moderate**	4	3	3	3	3	3	
**MG/ICF mild**	9	5	9	7	7	4	9
**MG/ICF moderate**	0	3	0	2	2	4	
**DG/ICF mild**	7	6	4	4	4	3	9
**DG/ICF moderate**	2	3	5	5	5	6	

*ICF (international classification of functioning and heath).

*EG*: easy group; *MG*: moderate group; *DG*: difficult group.

### Tasks and apparatus

All individuals performed the Simple Timing (ST) task and their performance was measured in milliseconds. The task was to touch a target in integration with a visual stimulus in such a way that the target would be touched simultaneously with the arrival of a light stimulus at the end of the runway. In order to perform the task, a coincident timing apparatus for complex tasks [31], which is described below, was used.

The apparatus is comprised of a 207 cm long, 10 cm wide, and 2 cm high runway. 96 diodes (LEDs) were placed in a straight line on the runway, 1 cm away from each other. The apparatus is also comprised of a 70 cm long, 40 cm wide, and 6 cm high wooden table on which a sensor was placed. In order to turn on the diodes in sequence, a computer with specific software was used, which was activated by the experimenter.

To perform the simple timing task, the subject sat in front of a table in front of his/her it was disposed an advice with a series of lights arranged in a row that linearly directed toward the participant. When the researcher activated the task, the lights began to illuminate sequentially, always from the farthest to the closest to the subject. The displacement of the light reached a speed of 18 meters per second. The goal of the subject was playing a sensor when the light that was closest to him was synchronously ignited. All subjects performed 20 consecutive attempts, each trial was processed in milliseconds by a specific software unit. From the data extracted by matching software the measurements were calculated. Those measurements present three measurements on the coincidence of the direction (advance or delay) and on the variability of performance, and the absolute error, variable and constant, respectively. The error constant is determined by the average performance of the blocks of trials, the absolute error is determined by the average absolute blocks of trials, and the error variable is determined by the average of the standard deviation of the blocks of each trial [19].

Data regarding basic skills are for sample characterization and items derived from the ICF - International Classification of Functioning and Health. It does not directly influence the performance results but illustrate more clearly the behavior of the sample in these items.

The items assessed were: acquisition of basic competences, acquisition of complex competences, attention and concentration, decision making, performance of complex tasks, and reporting and receiving messages. 1 is the score for slight difficulty and 2 for moderate difficulty.

### Data analysis

The results were analyzed in relation to absolute error, variable error, and constant error of each group. These measurements enable the verification of accuracy, consistence, and direction of the performance of each group, respectively.

The Levene's Test was used to assess the homogeneity of variances and it pointed out violation of the homogeneity assumptions for Absolute and Variable errors. Additionally, the Shapiro-Wilk test did not demonstrate the normality assumption in all errors assessed, which precluded the parametric analysis. Thus, in order to verify the performance difference among the groups, the Mann Whitney U test was applied. An alpha of 0.05 for statistical significance was adopted for all analyses.

## Results

**Figure 1:**
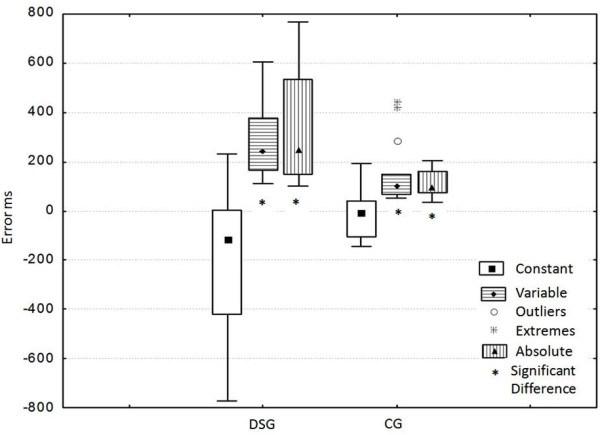
**Absolute, constant, and variable error (in milliseconds) obtained through the average of the 20 consecutive attempts performed by each individual.** DSG: Down Syndrome Group; CG: control group.

The Mann Whitney U test pointed out significant difference in the intergroup analysis for the accuracy adjustment of the motor action to the arrival of the visual stimulus at the end of the runway, that is, of the Absolute Error (Z = 3.656, p = 0.001); and for the performance consistence, which reflects in the Variable Error (Z = 2.939, p = 0.003). However, there was no difference among the groups regarding the performance direction, represented by the Constant Error (Z = −1.77, p = 0.07). These results are illustrated in Figure 1.

## Discussion

The aim of this study was to investigate the performance of individuals with DS in a simple Coincident Timing task, comparing them to healthy individuals. The initial hypothesis of the study was that the DS individuals would present impairment in the timing ability, therefore reflecting in slower and delayed responses.

The results of this experiment confirmed the hypothesis initially raised to the extent the individuals with DS presented a trend of delay in their motor responses, which corroborates the findings in the literature [32,33]. A possible explanation for these results may be in relation to the neural features presented by these individuals and their reflections in the motor and cognitive aspects, resulting in delay of the motor responses. This inconsistence in performance and inaccuracy when performing motor tasks that involve the time synchronization may be related to the brain stem, as DS individuals, when compared to individuals who do not have the syndrome, present such structure in a smaller size. Such dysfunction associated with the alteration in the cerebellum can justify the slow and delayed responses, as these structures are important for the time synchronization ability.

Based on our study, the absolute error allowed to observe that Down syndrome individuals have a poorer performance compared coincidentally matched individuals. Individuals with DS have greater difficulty performing the coincidence, when compared to controls, which denotes timing problems. According to Kandel (1997) [34] the cerebellum is responsible for coordinating the activity that needs accurate time occurrence, besides coordinating the planning, chronology, and the activity patterns of the skeletal muscles during the movement. Thus, cerebellar dysfunctions may lead to an increase in the time of reaction and abnormalities in the trajectory of the hands, evidencing even more its role of planning and regulation of the movement [35].

Considering the visual-motor system, we reported that the variable error suggests that individuals with Down syndrome tend to perform tasks in unstable and irregular coincidence of visual-motor systems, when compared to control subjects. The main problem of the DS individuals is in the deficit of the perceptual-motor abilities, which are responsible for the abilities that support the acquisition of several motor skills. This deficit can be justified by the structural features mentioned, which explain the difficulties these individuals have to acquire and improve motor skills [18,19,32].

Thus, as the coincident timing task depends on selection, planning, and execution processes to anticipate the arrival of the stimulus and perform the effector response simultaneously [22], these abilities are acquired as the perceptual-cognitive mechanism develops [20]. This fact can justify the delayed responses also presented by the control group, as there are studies that evidence greater accuracy in coincident timing tasks with the increase of age [36]. In this context, from 14 or 15 years old the performance curve in this type of task reaches an asymptote, thus making the anticipation ability equivalent to that of an adult [37]. When we observe the age of the individuals of the control group 20 (+/− 5 years old), some of them could be at the final stage of the perceptual-motor development, therefore justifying the delay noticed.

Another aspect to be highlighted is the amount of practice offered in the design of this study, which could explain the delay in responses, as the number of attempts was not enough to improve performance. Freudenheim (1994) [38] states that it is necessary to investigate whether the performance in a simple coincident timing task in adults is proportional to the number of attempts. It was observed that 90 attempts in a task of the same type were needed to ensure an optimum level of performance [39]. In this case, the delayed motor responses of the control group may be associated with the number of attempts offered. However, it should be noted that it is not a motor learning study, but an investigation of the performance in this type of task in a population with Down syndrome.

Nevertheless, as an important limitation of your study, the use of a mental age-matched control group in line with age is fundamental, as according to Sugden and Keogh (1990) [14] the DS individuals present a context of delay since the early phases of life and there is a tendency that such deficit increases over the years due to the reduction of experiences with time synchronization tasks, when compared to individuals who do not have the syndrome. The IQ of adult subjects with DS is approximately 40 to 70 points, while healthy adults are near 100 [40]. This difference should be accounted for when studying cognitive functions in DS.

A question to be answered refers to the impact of the alteration on the ability to perform timing tasks when performing daily activities. In this context, Gimenez, Stefanoni and Farias (2007) [18] state that individuals with DS can have a deficit in the motor ability regarding time synchronization and still be able to perform movement patterns that depend on such ability in a proper way or in a way that satisfactorily meets the environmental requirements throughout life, not reflecting in the deterioration of the performance of daily activities. However, this fact still needs future research.

It is known that, in manual tasks, the individuals with DS present characteristics such as slowness, selection of unusual strategies, and delay in the acquisition of key patterns of movement [17,33]. Another explanatory hypothesis for the slowness in movement is the fact that these individuals perform the movement slower in order to achieve more accuracy. This difference is due to the strategy adopted for motor control and therefore it is considered an adaptive reaction, and not necessarily a biological difference.

An important point in our study is important to be raised. Although we used a task that involves specific cognitive abilities, our main purpose was not to study the influence of cognitive status on the specific mentioned tests. We suggest a further experiment to specifically investigate this issue.

Our study presents important findings, since motor development has been recently investigated [41-44]. The task performed in this experiment has been criticized due to the fact that it is very simple for individuals that do not have the Down syndrome, which prevents the results achieved from being generalized for the performance of activities in the real world [26]. However, we believe it is a relevant task for individuals with DS, as time synchronization tasks are present in the daily routine of these individuals, such as crossing the street, entering the elevator, passing in the turnstile, climbing the escalator or even taking an object thrown by another person. In this sense, future studies can approach more complex tasks and with more ecologic validity, comparing them to more controlled tasks performed in laboratories, investigating the impact of the impairment on the time synchronization ability when performing daily activities.

## Conclusion

Individuals with DS, when compared to individuals of the same age who do not have the Syndrome, have more difficulty in integrating the motor action to an external stimulus and they also present more inconsistence in performance. However, even though the DS group had a higher level of delay, both groups presented the same tendency to delay their motor responses.

## Competing interests

We declare no conflict of interest.

## Authors’ contributions

CTP, GMGB, MAAS, GLA, GCSP, CBMM, LCA, VEV, APJ, RWW and UCC participated in the acquisition of data and revision of the manuscript. All authors conceived of the study, determined the design, interpreted the data and drafted the manuscript. CTP, VEV, LCA and UCC determined the design and drafted the manuscript. All authors read and gave final approval for the version submitted for publication.
